# Influencing factors on quality of life in women undergoing cancer treatment: a cross-sectional study

**DOI:** 10.4069/whn.2025.09.02

**Published:** 2025-09-30

**Authors:** Hyun Jin Jung, Yu Jin Hong, Seo Young Hong, Ju-Hee Nho

**Affiliations:** 1 Department of Nursing, Jeonbuk National University Hospital, Jeonju, Korea; 2 College of Nursing, Research Institute of Nursing Science, Jeonbuk National University, Jeonju, Korea

**Keywords:** Anxiety, Health status, Neoplasms, Physical functional performance, Quality of life

## Abstract

**Purpose:**

The purpose of this study was to identify factors affecting quality of life (QoL) in women undergoing cancer treatment, based on Ferrans’ QoL model, with particular attention to femininity and women’s roles.

**Methods:**

A cross-sectional correlational design was used, and 101 women were recruited through convenience sampling from a tertiary medical center in Jeonju, South Korea, between June and August 2024. QoL, death anxiety, performance status (ability to perform daily activities), body image, and division of household labor were measured using a structured questionnaire. Data were analyzed using the independent t-test, analysis of variance, Pearson correlation coefficients, and multiple regression analysis.

**Results:**

The majority of participants had either breast cancer (40.6%) or gynecologic cancer (26.7%). Participants’ QoL scores were at a moderate or higher level (69.12±16.65) and demonstrated statistically significant negative correlations with death anxiety (r=–.43, *p*<.001), performance status (r=–.44, *p*<.001), perceived health status (r=–.58, *p*<.001), and body image (r=–.46, *p*<.001). Performance status (β=–.30, *p*<.001), perceived health status (β=–.30, *p*<.001), and death anxiety (β=–.27, *p*=.001) were independent predictors of QoL in women with cancer during treatment (F=14.69, *p*<.001), with an overall explanatory power of 49.0%. Body image and division of household labor were not significant predictors of QoL.

**Conclusion:**

Performance status, perceived health status, and death anxiety should be prioritized when evaluating QoL in women undergoing cancer treatment.

## Introduction

According to 2022 Statistics Korea data, cancer is the leading cause of death among South Korean women, accounting for a substantial proportion of female mortality, with more than 80,000 cancer-related deaths annually [1]. Trends in cancer incidence since 2010 show increases in breast, endometrial, and ovarian cancers, which are primarily female-specific cancers. With improvements in survival rates, cancer is increasingly regarded as a chronic condition, underscoring the need for ongoing management and support for women following diagnosis [2,3].

While early detection, treatment, and management of side effects have long been the focus of cancer care, attention to the quality of life (QoL) of women with cancer is equally critical [4,5]. QoL is a multidimensional concept encompassing physical health, psychological well-being, independence, social relationships, and personal beliefs [6], and it serves as a key indicator of overall health in women with cancer [3]. Multiple factors influence QoL, including age, income, physical and emotional functioning, pain, fatigue, depression, and anxiety [7,8]. Studies have also shown that women with cancer report lower QoL than men with cancer [8,9].

The QoL model of Ferrans et al. [10], adapted from Wilson and Cleary [11], posits that QoL is shaped by biological function, symptoms, functional status, and general health perceptions, which influence QoL sequentially. Furthermore, the model emphasizes that individual and environmental characteristics indirectly affect QoL through these mediating factors.

Most women with cancer experience psychological symptoms such as anxiety, depression, and fear after diagnosis, frequently accompanied by death anxiety [12-14]. Women, in particular, tend to experience greater death anxiety [15], which has been shown to negatively affect QoL and exert profound effects on physical and psychological health as well as disease prognosis [16]. Death anxiety has been identified as a predictor of QoL in women with cancer, and evidence suggests that reducing death anxiety may improve QoL [17]. However, previous studies have primarily focused on patients with lung [18] or breast cancer [19], and gender-specific analyses remain limited.

Functional status, defined as the ability to perform daily activities such as self-care, work, and leisure [20], is also known to influence both overall health and QoL in patients with cancer [21,22]. Functional status may differ by gender, yet few studies have examined these differences. Moreover, performance status is an important consideration in prognosis, treatment selection, treatment outcomes, and rehabilitation strategies for patients with colorectal cancer [23]. Managing performance status is therefore essential for improving QoL in women with cancer.

Perceived health status is another widely used indicator of overall health, as it strongly influences QoL [24,25]. Prior studies have found that cognitive factors, such as perceived health status, are significantly associated with QoL in patients with cancer [26]. Consequently, identifying and improving health perception plays a critical role in maintaining higher QoL in women with cancer.

Beyond physical and psychological challenges, women often face additional QoL burdens related to gender-specific issues, including concerns about femininity [27,28] and household labor [29]. Postoperative wounds, as well as the loss of breasts, uterus, or ovaries due to surgery, chemotherapy, or radiation therapy, negatively affect body image and may diminish self-control, which can in turn affect adherence to treatment [30,31]. Body image, a key aspect of sexual health, is often significantly diminished after treatment in women with cancer [27]. Compared with men, women with cancer report greater body image concerns [32,33], which have been shown to negatively impact QoL [28]. These findings underscore the importance of addressing body image issues in cancer care.

Women with cancer also devote significantly more time to household labor than men with cancer [34]. Studies report that married patients with cancer have worse QoL than unmarried patients [9,35]. Unmarried women with cancer often face economic challenges and limited access to social or policy support, whereas married women experience additional burdens from household labor and balancing employment responsibilities [29,36]. Household labor, which disproportionately falls on women, is associated with increased depression and reduced life satisfaction [37]. These findings highlight the need to investigate how the division of household labor affects the QoL of women with cancer.

Although many studies have examined factors influencing QoL in women with cancer, most have emphasized physical and psychological variables [5,7,38]. Few have incorporated femininity and women’s roles into analyses of QoL in women undergoing cancer treatment.

Therefore, this study aimed to identify factors influencing QoL in women with cancer based on Ferrans’ QoL model [10], including femininity and women’s roles. The specific objectives were as follows:

(1) To examine whether QoL differs according to the general characteristics of women with cancer undergoing treatment.

(2) To identify correlations among QoL, death anxiety, performance status, perceived health status, body image, and division of household labor.

(3) To determine the factors that influence the QoL of women with cancer during treatment.

## Methods

**Ethics statement**: This study was approved by the Institutional Review Board of Jeonbuk National University Hospital (No. IRB-2024-04-044-001). Informed consent was obtained from the participants.

### Study design

This correlational study employed a cross-sectional survey design. The study adhered to the STROBE (Strengthening the Reporting of Observational Studies in Epidemiology) reporting guidelines (http://www.strobe-statement.org) in the description of methods and findings.

### Participants

Participants were women with cancer admitted to the wards of a tertiary general hospital in Jeonju, South Korea. The inclusion criteria were: (1) women aged 19 years or older; (2) diagnosed with any type of solid cancer, stages I–IV; (3) currently undergoing cancer treatment (chemotherapy, radiation therapy, surgical treatment, and/or antihormonal therapy, including a 5-year course of tamoxifen for breast cancer); (4) having a spouse; and (5) understanding the purpose of the study and voluntarily consenting to participate. The exclusion criteria were: (1) patients with hematologic malignancies, (2) patients with stage 0 cancer, and (3) individuals self-reporting the use of psychiatric medication for depression, anxiety, or other psychiatric disorders since their cancer diagnosis. Hematologic malignancies were excluded because, as systemic diseases, their progression, staging, physical symptoms, treatment modalities, and prognoses differ substantially from those of solid tumors. These differences may affect the factors influencing QoL; thus, the exclusion was applied to ensure homogeneity in the study sample and enhance the validity of the analysis [39-41].

The sample size was estimated using G*Power ver. 3.1.9.7 (Heinrich Heine University Düsseldorf, Düsseldorf, Germany). For regression analysis with a medium effect size of 0.15 [42], five independent variables were included (death anxiety, performance status, perceived health status, body image, and division of household labor). With a significance level of 0.05 and power of 0.80, the minimum required sample size was 98. Data from 110 participants were collected, and nine were excluded due to incomplete questionnaires (≥50% missing responses). Thus, 101 participants (91.8%) were included in the final analysis, which was deemed sufficient.

### Measurements

#### Quality of life

QoL was assessed using the Korean version [43] of the Functional Assessment of Cancer Therapy–General version 4 (FACT-G) [44], originally developed by Cella et al. [45]. The instrument consists of 27 items, including seven on physical well-being, seven on social well-being, six on emotional well-being, and seven on functional well-being. Responses are rated on a 5-point Likert scale from 0 (“strongly disagree”) to 4 (“strongly agree”). The total score ranges from 0 to 108, with higher scores reflecting better QoL. Cronbach’s α was .92 in the original study [45], .89 in the Korean version [43], and .86 in this study.

#### Death anxiety

Psychological symptoms were measured using the Death Anxiety Scale developed by Templer [46], adapted and validated for Korean populations by Ko et al. [47]. The tool consists of 15 items: five on fear of death itself, two on denial of death-related thoughts, two on perception of limited time, and six on fear of death-related events. Responses are rated on a 5-point Likert scale from 1 (“strongly disagree”) to 5 (“strongly agree”). Scores range from 15 to 75, with higher scores indicating greater death anxiety. Cronbach’s α was .83 in Templer’s study [46], .80 in Ko et al. [47], and .82 in this study.

#### Performance status

Functional status was assessed using the Performance Status Scale of the Eastern Cooperative Oncology Group [20]. This single-item measure ranges from 0 (“able to perform all normal activities”) to 4 (“completely bedridden and unable to care for oneself”). Lower scores indicate better ability to perform daily activities.

#### Perceived health status

Perceived health status was assessed using a single item from the Korea National Health and Nutrition Examination Survey, developed by the Korea Disease Control and Prevention Agency [48]. Responses range from 1 (“very good”) to 5 (“very bad”), with higher scores indicating worse perceived health.

#### Body image

Body image, reflecting femininity as an individual characteristic, was measured using the scale developed by Hopwood et al. [49], translated and adapted for Korean patients with cancer by Kim et al. [50]. The tool consists of 10 items, including five on overall body image and five on cancer-specific experiences. Items are rated on a 4-point Likert scale from 0 (“strongly disagree”) to 3 (“strongly agree”). Scores range from 0 to 30, with higher scores indicating greater body image disturbance. Cronbach’s α was .93 in the original study [49], .91 in a Korean study on breast cancer [50], and .90 in this study.

#### Division of household labor

The Division of Household Labor scale developed by Kim and Koh [51] was used to assess women’s roles as an environmental characteristic. The scale includes 18 items: five on dietary habits, three on clothing, four on housing and living environment, three on shopping and home management, and three on caregiving. Response options range from 0 (“not applicable”), 1 (“mostly done by the husband”), to 5 (“mostly done by the wife”). Total scores range from 0 to 90, with higher scores indicating greater responsibility for household labor by the wife. Cronbach’s α was .91 at development [51] and .87 in this study.

### Data collection

Data were collected between June 1 and August 14, 2024. Questionnaires were distributed to eligible hospitalized women after permission was obtained from the nursing department, the attending physician, and the head nurse. Recruitment was announced through a ward bulletin board notice. Questionnaires were administered directly by the researcher and completed in either the ward conference room or a private space. Participants returned completed questionnaires in sealed envelopes. Each questionnaire required approximately 20–30 minutes, and participants received a small gift (approximately 4 US dollars in value) as a token of appreciation.

### Data analysis

Data were analyzed using IBM SPSS ver. 29.0 (IBM Corp., Armonk, NY, USA). Descriptive statistics were used to summarize participants’ general characteristics and main variables. The independent t-test and one-way analysis of variance were used to examine differences in variables by general characteristics, with post hoc analyses performed using the Scheffé test. Pearson’s correlation coefficients were calculated to examine relationships among variables. Multiple regression analysis was conducted to identify factors influencing QoL.

## Results

### Differences in quality of life according to general characteristics

The mean age of participants was 55.29±10.13 years, with the largest group being those under 49 years of age (n=33, 32.7%). In terms of education, 44 participants (43.6%) had a university degree or higher. Most participants (n=61, 60.4%) were unemployed, and 67 participants (66.3%) reported having a religion. The largest proportion of participants had a monthly income between 2 million and 3 million Korean won (24.7%). Breast cancer was the most common diagnosis (n=41, 40.6%), followed by gynecologic cancer (n=27, 26.7%). The average time since cancer diagnosis was 19.48±37.84 months, with most participants (n=69, 68.4%) diagnosed within the past 12 months. Stage II was the most frequent cancer stage (n=27, 26.7%), and 74 participants (73.3%) had no recurrence. Chemotherapy was the most common current treatment (n=58, 57.4%). QoL differed significantly by educational level; post hoc analysis showed that participants with a university degree or higher reported higher QoL compared with those with only a high school education (F=3.97, *p*=.022). In addition, women without recurrence had significantly higher QoL than those with recurrence (t=2.98, *p*=.004) (Table 1).

### Levels of quality of life, death anxiety, performance status, perceived health status, body image, and division of household labor

The mean QoL score of participants was at a moderate or higher level (69.12±16.65). Death anxiety was at a moderate level (45.66±10.10). The mean performance status score was 0.93±0.71, indicating a relatively low level. Perceived health status was at a moderate or lower level (3.05±0.84), body image was also moderate or lower (12.62±6.36), and the division of household labor was moderate (49.28±14.96) (Table 2).

### Correlations among quality of life, death anxiety, performance status, perceived health status, body image, and division of household labor

QoL scores showed statistically significant negative correlations of moderate magnitude with death anxiety (r=–.43, *p*<.001), performance status (r=–.44, *p*<.001), perceived health status (r=–.58, *p*<.001), and body image (r=–.46, *p*<.001) (Table 3).

### Factors affecting quality of life

Tolerance values ranged from 0.66 to 0.87 (all >0.1), and variance inflation factor values ranged from 1.14 to 1.51 (all <10), indicating no multicollinearity among the independent variables. The Durbin-Watson statistic was 2.26, confirming no autocorrelation in the residuals. Regression analysis included variables that showed significant differences in QoL based on general characteristics (education level and cancer recurrence) and variables significantly correlated with QoL (death anxiety, performance status, perceived health status, and body image). Results revealed that death anxiety (β=–.27, *p*=.001), performance status (β=–.30, *p*<.001), and perceived health status (β=–.30, *p*<.001) were significant predictors of QoL. In other words, lower death anxiety, higher performance status, and better perceived health were associated with greater QoL. These variables together explained 49.0% of the variance in QoL among women with cancer (Table 4).

## Discussion

This study aimed to examine the factors influencing the QoL of women with cancer undergoing treatment based on the QoL model [10], with consideration of femininity and gender roles. The results demonstrated that performance status, perceived health status, and death anxiety significantly affected participants’ QoL.

Among these variables, performance status and perceived health status exerted the greatest influence on QoL in women undergoing treatment. These findings are consistent with previous research [52], which showed that women with cancer and those with poorer performance status tend to report lower QoL compared to their counterparts. A higher performance status allows patients to accomplish desired tasks more effectively, maintain independence in daily activities, and participate productively in society, thereby enhancing QoL. However, cancer patients frequently experience physical limitations due to treatment-related side effects, cancer-related fatigue, pain, muscle weakness, lymphedema, and surgery [53]. Prior studies indicate that only 31.3% to 49.6% of cancer survivors meet recommended levels of physical activity [54]. Therefore, individualized monitoring of activity levels and the application of tailored intervention programs using both online strategies (e.g., mobile apps, wearable devices) and offline methods (e.g., group exercise, individualized counseling) are essential to promote physical activity in women with cancer. Such multifaceted approaches are expected to meaningfully increase participation in physical activity.

This study also found that better perceived health status was associated with higher QoL, consistent with findings in patients with breast [5,55] and cervical cancer [35,56], where those who rated their health status as good or moderate reported higher QoL scores. Similar results have been reported in studies involving adult cancer patients, where subjective health status was identified as a major determinant of QoL [7]. These findings suggest that subjective health perceptions vary depending on individual circumstances and that a positive perception of health can substantially enhance QoL. Accordingly, it is necessary to develop and implement individualized nursing interventions that assess and improve patients’ perceptions of their health. Such interventions should incorporate comprehensive evaluations of factors including age, cancer type, treatment stage, and psychosocial characteristics. Strategies may include personalized health education, psychological counseling, exercise and nutrition programs, and family participation initiatives. Additionally, continuous monitoring and individualized feedback delivered through mobile applications or telephone counseling may further support improvements in patients’ health perception and overall QoL.

Death anxiety was identified as the third most significant factor influencing QoL in women with cancer undergoing treatment. This finding is consistent with previous studies reporting that higher death anxiety is associated with lower QoL among women with breast or other solid cancers, including gastric cancer, in Iran [15,17]. One study further noted that Iranian women with cancer experienced higher death anxiety and lower QoL than men [15]. A cancer diagnosis imposes a considerable burden on patients, negatively affecting physical, psychological, and social domains [56]. In particular, women with cancer have been reported to experience greater death anxiety and lower QoL than men, indicating that gender is an important determinant of QoL in patients with cancers of the gastrointestinal tract, breast, head and neck, and other sites [16]. Additionally, both death anxiety and psychological distress have been identified as factors contributing to reduced QoL in women with breast and other solid cancers, including gastric cancer [17]. To reduce death anxiety, continuous psychological and professional counseling, structured support programs specifically targeting death-related concerns, and family education initiatives are essential. Such approaches can foster mutual understanding and support within families, ultimately alleviating death anxiety and improving QoL for women with cancer. By contrast, body image and division of household labor were not significantly associated with QoL in this study. This differs from some prior studies showing that lower body image is linked to poorer QoL [57,58] and that sharing household chores and receiving family support positively influence QoL [59]. However, the present findings are consistent with qualitative research on breast cancer patients in their 50s, which reported that the division of household labor did not significantly affect QoL [29]. This discrepancy highlights the potential impact of cancer stage, treatment status, and family dynamics on QoL outcomes. Studies of breast cancer survivors have found that a positive body image improves QoL [58], while research on women undergoing treatment or declared cancer-free emphasized the importance of spousal physical and emotional support, as well as cooperation in household responsibilities, for improving QoL [59]. For women with cancer, femininity and gender roles within the household may be perceived as burdens that influence QoL. However, the absence of such associations in this study may reflect the characteristics of the sample. Specifically, 68.4% of participants had been diagnosed within the past year, suggesting that most were actively engaged in treatment and prioritized immediate health concerns over body image or household responsibilities. This study also included a broader range of cancer types beyond breast cancer, such as gynecologic, thyroid, and lung cancer. The impact of cancer type may explain differences in how body image and femininity influence QoL. Breast and gynecologic cancers have more direct implications for femininity, whereas other cancers may not immediately affect body image or social roles. Furthermore, shifts in family role distribution often occur after a cancer diagnosis, and in advanced disease situations, recovery, emotional support, and treatment needs may outweigh issues related to femininity or household labor. Thus, the significance of this study lies in its identification of complex and interrelated factors—namely, cancer type, treatment timing, and family role changes—that shape QoL. Future longitudinal studies are needed to explore femininity and gender roles among women with cancer across different stages of survivorship.

This study has several limitations. It was conducted in a single institution in one region, which restricts the generalizability of the findings. Additionally, the inclusion of breast cancer patients undergoing long-term tamoxifen therapy may have introduced time-related differences in treatment experiences. Approximately 20% of participants did not know their cancer stage, which could have affected self-reported data. Moreover, the instrument used to measure household labor division was originally developed for women with retired husbands, with 52.9% of participants aged 60 years or older [51]. In contrast, the mean age in this study was 55.29 years, and most participants were relatively younger women living with their spouses. Therefore, the tool may not have been fully appropriate for this population. Future research should refine the concept of household labor in women with cancer and develop a more suitable measurement instrument.

In conclusion, this study contributes to the literature by identifying factors influencing the QoL of women undergoing cancer treatment using a QoL model that incorporated femininity and gender roles. Performance status, perceived health status, and death anxiety were identified as significant determinants of QoL, together explaining 49% of the variance. Nurses play a pivotal role in improving QoL by addressing modifiable factors through clinical practice. Priority areas include promoting functional capacity through individualized rehabilitation strategies, reinforcing health perception via ongoing assessment and patient education, providing psychosocial support to reduce death anxiety, and integrating spiritual care into routine practice. These strategies underscore the importance of comprehensive, patient-centered nursing care in improving QoL among women with cancer.

## Figures and Tables

**Table 1.** Differences in quality of life according to general characteristics (N=101)

Characteristic	Categories	Mean±SD or n (%)	Quality of life		
			Mean±SD	r or t or F	*p*
Age (year)		55.29±10.13		–.11	.297
	≤49	33 (32.7)	71.55±14.74	0.55	.648
	50–59	32 (31.7)	68.56±18.22		
	60–69	25 (24.8)	68.76±18.72		
	≥70	11 (10.8)	64.27±12.61		
Educational level				3.97	.022
	≤Middle school	20 (19.8)	65.50±17.38^a^		(b<c)^†^
	High school	37 (36.6)	64.95±15.84^b^		
	≥University	44 (43.6)	74.27±15.90^c^		
Occupation				–1.69	.094
	No	61 (60.4)	66.87±17.20		
	Yes	40 (39.6)	72.55±15.34		
Religion				0.23	.822
	No	34 (33.7)	69.65±16.78		
	Yes	67 (66.3)	68.85±16.70		
Monthly income (million Korean won)^‡^				2.11	.085
	<1	24 (23.7)	61.46±17.92		
	1–1.99	15 (14.9)	71.27±14.69		
	2–2.99	25 (24.7)	68.56±13.81		
	3–3.99	15 (14.9)	72.87±19.91		
	≥4	22 (21.8)	74.09±15.33		
Type of cancer				1.00	.414
	Breast cancer	41 (40.6)	68.02±15.88		
	Gynecologic cancer^§^	27 (26.7)	66.04±16.47		
	Gastrointestinal cancer^ǁ^	21 (20.8)	71.33±17.02		
	Lung cancer	8 (7.9)	73.50±18.52		
	Thyroid cancer	4 (4.0)	80.75±20.65		
Time since diagnosis (month)		19.48±37.84		.35	.704
	<12	69 (68.4)	69.93±15.56		
	12–35	16 (15.8)	66.06±19.56		
	≥36 (range, 36–288)	16 (15.8)	68.69±18.81		
Stage of cancer				0.97	.427
	I	20 (19.8)	73.75±14.63		
	II	27 (26.7)	67.81±17.10		
	III	19 (18.8)	63.95±14.68		
	IV	15 (14.9)	68.93±15.35		
	Unknown	20 (19.8)	71.30±20.25		
Cancer recurrence status				2.98	.004
	No	74 (73.3)	71.99±15.37		
	Yes	27 (26.7)	61.26±17.74		
Type of treatment				2.81	.065
	Operation only	33 (32.7)	70.52±17.31		
	Chemotherapy	58 (57.4)	66.55±15.63		
	Others^¶^	10 (9.9)	79.40±17.29		

† Scheffé test.

‡ One million Korean won is approximately 750 US dollars.

§ Gastrointestinal cancer: colorectal cancer, common bile duct cancer, gastric cancer.

ǁ Gynecologic cancer: cervical cancer, endometrial cancer, ovarian cancer, vaginal cancer.

¶ Others: antihormonal therapy, chemotherapy + operation, radiation therapy, radiation therapy + antihormonal therapy.

**Table 2.** Levels of quality of life, death anxiety, performance status, perceived health status, body image, and division of household labor (N=101)

Variable	Mean±SD	Data range	Possible score range
Quality of life	69.12±16.65	30–106	0–108
Physical well-being	19.01±6.07	2–28	0–28
Social/family well-being	17.54±5.69	4–28	0–28
Emotional well-being	16.04±5.25	3–26	0–24
Functional well-being	16.48±6.81	2–28	0–28
Death anxiety	45.66±10.10	22–70	15–75
Performance status	0.93±0.71	0–3	0–4
Perceived health status	3.05±0.84	2–5	1–5
Body image	12.62±6.36	0–25	0–30
Division of household labor	49.28±14.96	16–75	0–90

**Table 3.** Correlations among study variables (N=101)

Variable	r (*p*)				
	Quality of life	Death anxiety	Performance status	Perceived health status	Body image
Quality of life	1				
Death anxiety	–.43 (<.001)	1			
Performance status	–.44 (<.001)	–.06 (.586)	1		
Perceived health status	–.58 (<.001)	.32 (.001)	.31 (.002)	1	
Body image	–.46 (<.001)	.43 (<.001)	.20 (.043)	.43 (<.001)	1
Division of household labor	.09 (.355)	.19 (.054)	–.21 (.033)	–.19 (.057)	–.01 (.957)

**Table 4.** Factors influencing quality of life (N=101)

Variable	B	SE	β	t	*p*
Constant	120.90	6.40		18.90	<.001
Educational level^†^					
≤Middle school	–1.69	3.43	–.04	–0.49	.625
High school	–2.51	2.79	–.07	–0.90	.371
Cancer recurrence status^†^	–2.91	2.86	–.08	–1.02	.311
Death anxiety	–0.45	0.14	–.27	–3.31	.001
Performance status	–7.10	1.89	–.30	–3.75	<.001
Perceived health status	–5.98	1.74	–.30	–3.44	<.001
Body image	–0.35	0.22	–.13	–1.56	.123
R²	.53				
Adjusted R²	.49				
F (*p*)	14.69 (<.001)				

† The reference values were as follows: educational level (0=university and more), cancer recurrence status (0=no).
